# An Anaerobic Culture Study to Assess the Prevalence of Porphyromonas gingivalis in Periodontal Disease Incidences Among Adults

**DOI:** 10.7759/cureus.65023

**Published:** 2024-07-21

**Authors:** Dhanasree K Balan, Jiji John, Shabeer Ahamed, Nita Syam, Greeshma Sudhakaran, Lakshmy M, Hasbeena Ali

**Affiliations:** 1 Department of Periodontology, Malabar Dental College and Research Centre, Malappuram, IND

**Keywords:** periodontology, porphyromonas gingivalis, periodontitis, caries, anaerobic culture

## Abstract

Introduction

Periodontitis is a complex condition influenced by various factors involving interactions between the host and bacterial plaque. *Porphyromonas gingivalis*, an anaerobic gram-negative bacterium, is commonly linked with periodontal disease.

Aim

This study aimed to examine the occurrence of *P. gingivalis* in individuals diagnosed with chronic periodontitis (CP) compared to those who show no clinical indications of periodontal disease.

Methodology

Patients diagnosed with CP (including both severe and moderate cases) and individuals without any signs of periodontal disease were recruited for this study. Samples were collected from the gingival pockets using curettes and were subsequently subjected to anaerobic culturing.

Results

A group of 30 patients, divided into moderate and severe CP, along with 30 healthy individuals serving as controls, were examined. In individuals with CP, *P. gingivalis* was found in 23 (78%) of cases, while in healthy individuals, the prevalence was 10 (34%). The presence of *P. gingivalis* was notably higher in those with periodontal diseases compared to healthy subjects, with rates of 23 (78%) vs. 10 (34%), respectively.

Conclusion

*P. gingivalis* is frequently found in individuals with periodontal diseases as well as in those without such conditions, albeit in smaller quantities. Consequently, the existence of *P. gingivalis* raises the probability of developing periodontal disease and may be regarded as a notable potential contributor to its initiation.

## Introduction

Periodontitis, a chronic and multifactorial inflammatory condition, is linked with dysbiotic plaque biofilms. This long-lasting inflammatory disease commonly affects both soft and hard tissues surrounding the teeth worldwide [1]. The onset of gingival inflammation, triggered by bacterial biofilm formation, is marked by the degradation of supportive tooth tissues, including the periodontal ligament and alveolar bone. This process culminates in gum recession, damage to soft tissue, deterioration of bone structure, and, eventually, loss of teeth. Furthermore, it poses a significant risk for various systemic diseases like rheumatoid arthritis, heart disease, high blood pressure, diabetes, respiratory issues, and complications during pregnancy later in life [2-3].

The seriousness and progression of periodontal disease are affected by different risk elements, such as genetic predisposition, environmental factors, and characteristics related to the host. The bacteria associated with periodontitis gather in the sub-gingival plaque, largely made up of gram-negative strict anaerobic rods. Within the multitude of bacterial species residing in the oral cavity, there exists a bacterial cluster referred to as the "red complex," comprising *Porphyromonas gingivalis*, *Treponema denticola*, and *Tannerella forsythia*, which have been significantly associated with advanced periodontal damage [4-7].

Moreover, as indicated by various published studies, additional species, including *Fusobacterium *spp., *Prevotella *spp., *Campylobacter rectus*, *Eubacterium nodatum*, and *Aggregatibacter actinomycetemcomitans* have been found to be closely linked with periodontal disease [2]. *P. gingivalis* plays a vital role as the central pathogen in disrupting the balance of the oral microbiome and initiating periodontitis. It is a gram-negative, anaerobic bacterium with a rod-shaped structure, forming dark colonies on blood agar. This pathogen has been identified in around 85.75% of subgingival plaque samples collected from individuals suffering from chronic periodontitis (CP).

The occurrence of *P. gingivalis* is closely linked to the intensity of periodontal disease and is acknowledged as one of the primary factors contributing to the development of periodontitis. As per the keystone pathogen theory, even in small quantities, *P. gingivalis* can trigger CP by altering the balance of the normal bacterial community, leading to a state of dysbiosis that eventually leads to the initiation of the disease. *P. gingivalis* disrupts the host immune system by producing several virulence factors, including lipopolysaccharide, fimbriae, capsule, proteolytic enzymes, and various other surface structures/ligands [8]. Virulence factors are crucial substances produced by an organism throughout its life cycle, capable of harming the host [9]. The surface elements of *P. gingivalis* promote the creation of a biofilm, providing protection for the bacterium against the host's immune defenses [10,11].

Because of the widespread occurrence and potential complications associated with periodontal disease, it has become imperative to establish treatment plans aimed at disease prevention. A significant component of periodontal disease prevention involves identifying potential periodontal pathogens and assessing the risk factors that contribute to the incidence of periodontal disease. These data are vital for the formulation of preventive and control tactics [12].

Since *P. gingivalis* is identified as a notable causative factor and risk element for periodontal disease, it is imperative to examine the prevalence of periodontal pathogens using specific culturing techniques [13]. Microbiological laboratory protocols are pivotal in diagnosing and monitoring periodontitis therapeutically. Microbiological results act as significant prognostic indicators, forecasting the stability of attachment gain. It is noteworthy that numerous of these microorganisms may also be present in individuals with periodontal health and can coexist harmoniously with the host [14]. The current study aimed to determine the prevalence of *P. gingivalis* in patients diagnosed with CP and in individuals considered healthy, utilizing anaerobic culturing techniques.

## Materials and methods

The participants in this study were chosen from the Department of Periodontology at Malabar Dental College and Research Centre in Kerala. The study included 30 consecutive patients diagnosed with CP, all demonstrating clinical and radiographic signs of alveolar bone loss and periodontal pockets measuring 5 mm or more in-depth. Additionally, 30 periodontally healthy individuals without any clinical signs of periodontal disease were chosen. None of the periodontal patients or healthy participants had used antibiotics in the three months leading up to sample collection. Both groups were matched in terms of age and gender. Written consent was procured from all participants for their participation in the study. The ethical approval was obtained from Malabar Dental College and Research Centre with reference number MDCRC/IEC/2021/235.

**Figure 1:**
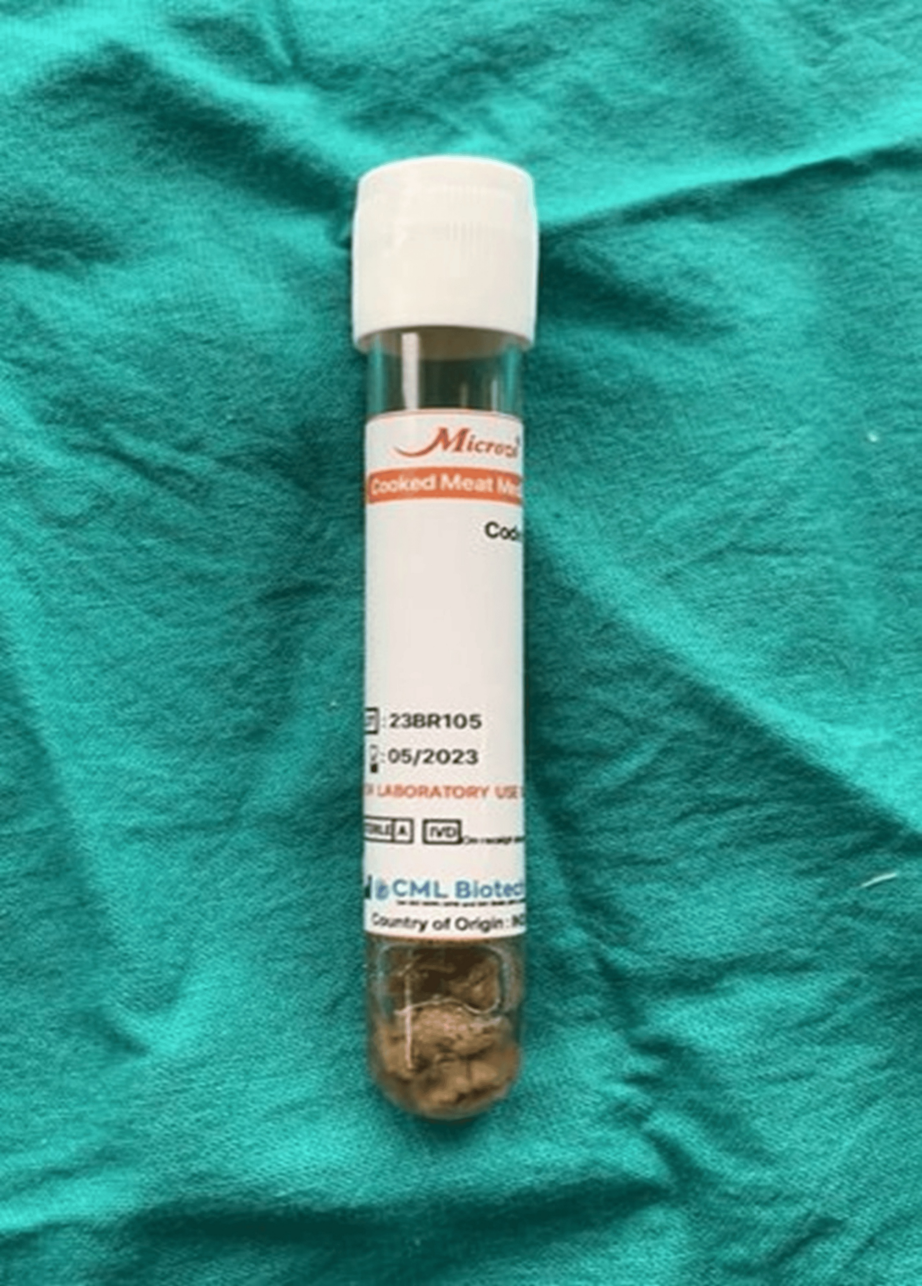
Robertson cooked meat medium for sample collection

**Figure 2:**
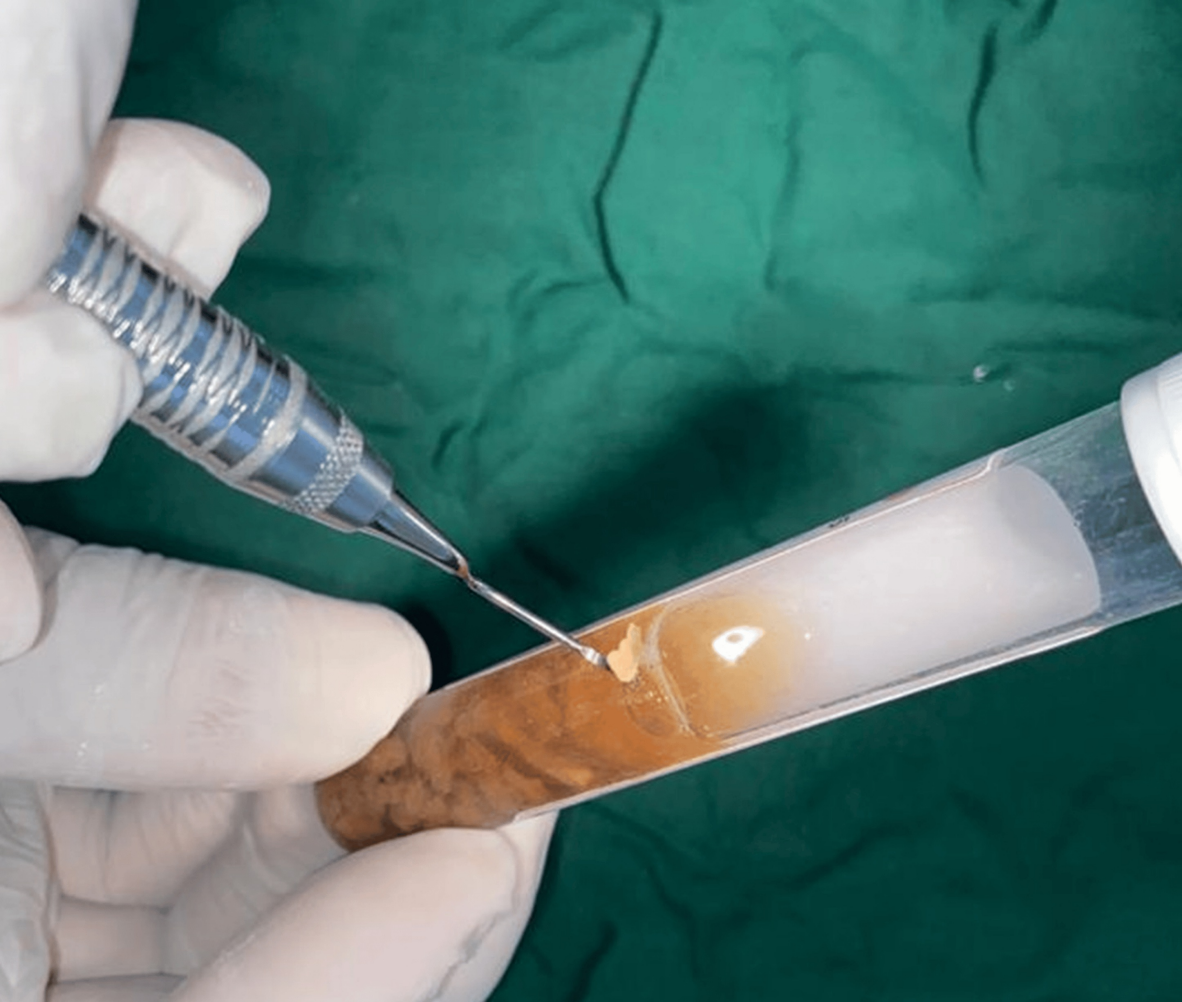
Samples were transferred into Robertson cooked meat medium

Samples were collected in stringent sterile environments. Initially, tooth surfaces were dried with sterile gauze to prevent contamination from saliva. Subgingival plaque samples were obtained from pockets/areas with densely populated periodontal bacteria using a sterile periodontal Gracey curette. These samples were subsequently placed in Robertson's cooked meat medium and promptly conveyed to the microbiology laboratory for immediate processing (Figure 1 and Figure 2).

**Figure 3:**
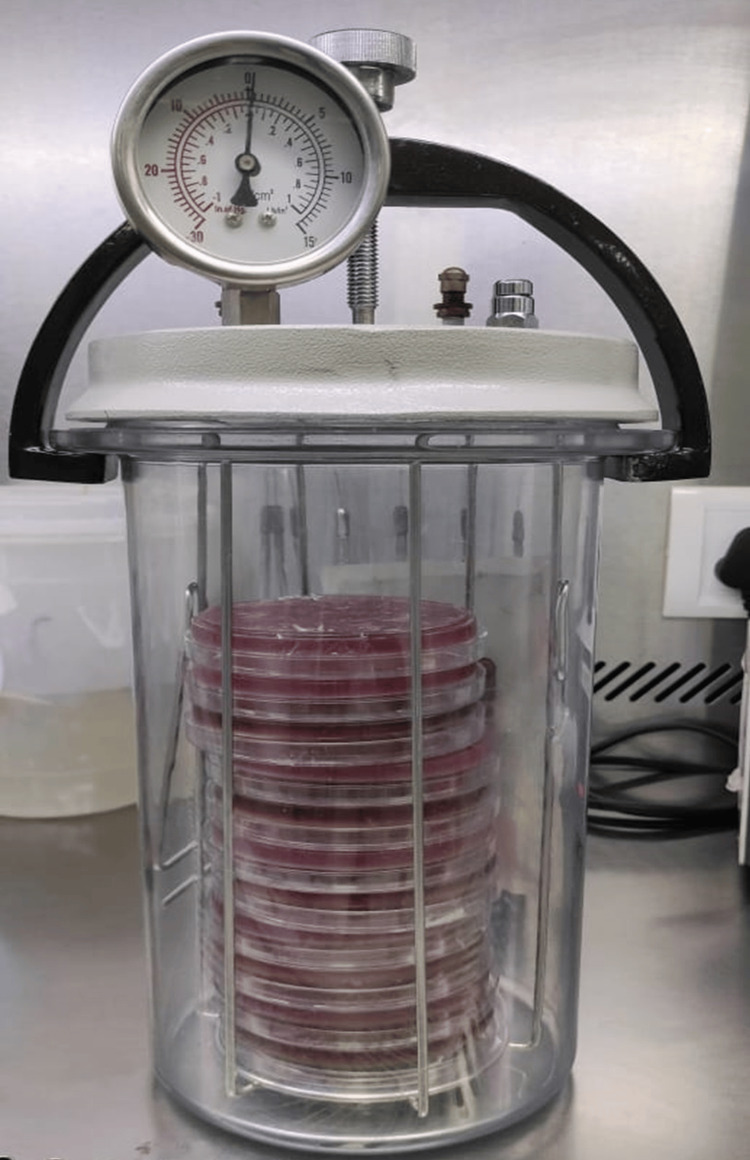
Plate incubated in an anaerobic jar

The GasPak system (Becton Dickinson Microbiology Systems, Gurgaon, India) creates an environment devoid of oxygen. It comprises a 2.5-L jar housing palladium catalyst pellets and a GasPak anaerobic envelope. Before each application, the pellets were heated in a 125°C oven for two hours. To activate the GasPak anaerobic envelope before incubating blood agar plates, 10 mL of water was introduced into the envelope. This procedure guaranteed a final concentration of carbon dioxide (CO_2_) ranging from 4% to 10%. Anaerobic conditions were assessed during the 60-minute incubation period using disposable anaerobic indicator strips. These strips change color based on the oxygen concentration, indirectly confirming the presence of CO_2_ (Figure 3).

**Figure 4:**
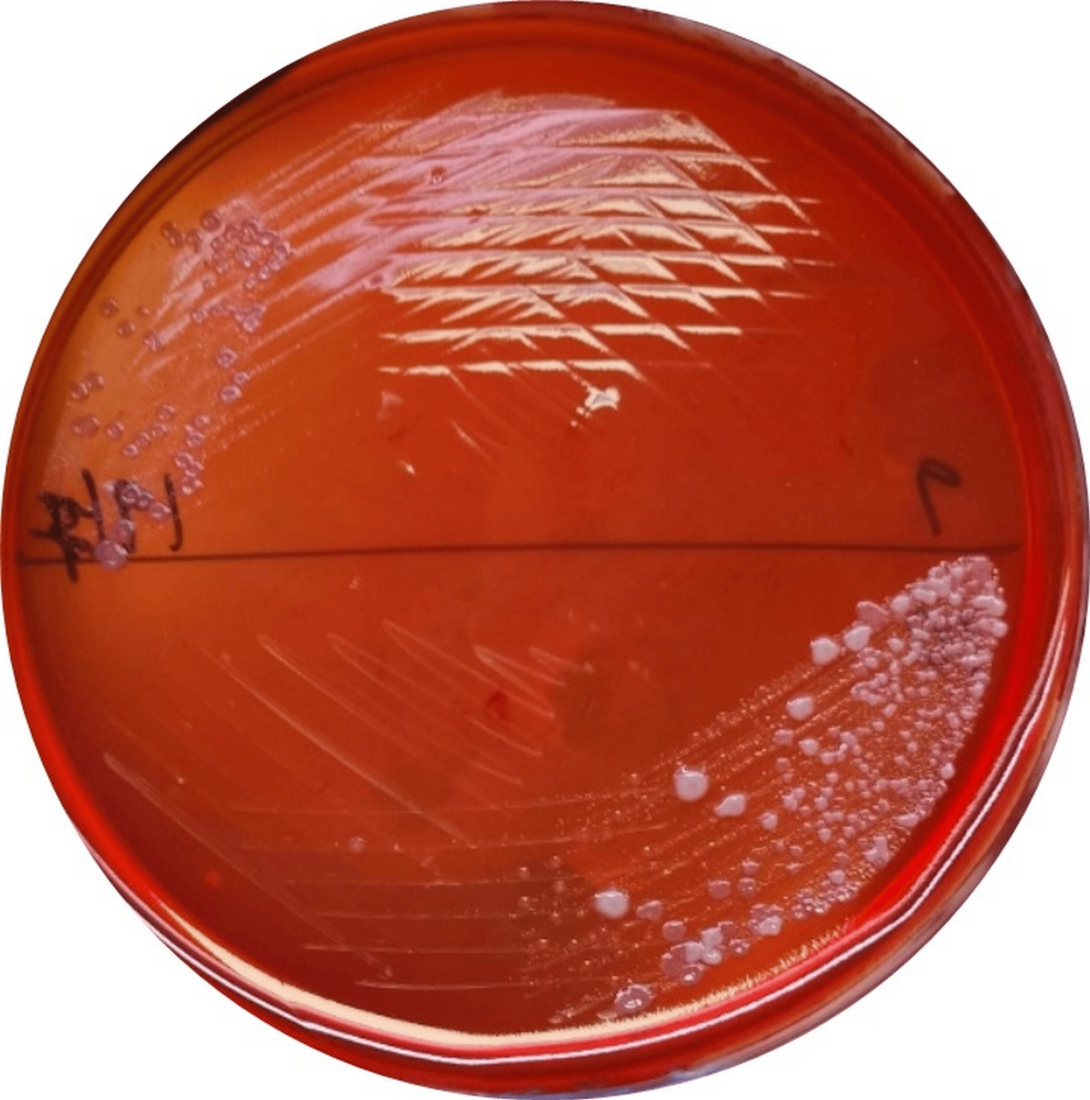
Colonization of Porphyromonas gingivalis

In the laboratory, the sample in Robertson's cooked meat medium was transferred and inoculated onto both blood agar. Subsequently, the samples were incubated at 37°C under anaerobic conditions. Anaerobic cultural methods are used to understand the oral microbiota of periodontitis. The moderate inflammation CP group was dominated by a complex of *P. gingivalis*, and by culturing, *P. gingivalis* grows as black-pigmented colonies on blood agar. It is cultured under a reducing atmosphere in enclosed cabinets or the GasPak method. A biochemical test oxidase was done to confirm *P. gingivalis* morphologically (Figure 4).

The data were analyzed using the statistical software Statistical Package for the Social Sciences (IBM SPSS Statistics for Windows, IBM Corp., Version 26.0, Chicago, IL), with a significance level set at p < 0.05. Descriptive statistics were used to assess the median and interquartile range of bacterial counts in each group. The normality of the data was checked using the Shapiro-Wilk test. Inferential statistics, specifically the Mann-Whitney U test, were performed to determine any discrepancies between the groups.

## Results

The comparison of bacterial counts between the CP and healthy people (HP) groups reveals that there was a significant difference in median count between the two groups. The median bacterial count for the CP group is substantially higher at 25,750 with an interquartile range of 20,000-42,000, compared to the HP group's median of 8,000 with an interquartile range of 4,500-18,000 (Table 1).

**Table 1:** Distribution of bacterial count among groups N: Number of participants

Group	N	Bacterial count	
		Median	Interquartile range
Chronic periodontitis	30	25,750	20,000-42,000
Healthy people	30	8,000	4,500-18,000

The Mann-Whitney U test further supports these observations, revealing a statistically significant difference between the groups (p < 0.001), with the CP group displaying a mean rank of 41.4 compared to the HP group's mean rank of 19.6. The substantial effect size, as indicated by the Z-value of 4.859, underscores the statistical significance of the observed differences in bacterial counts between the CP and HP groups (Table 2).

**Table 2:** Comparison of bacterial count between groups N: Number of participants

Group	N	Mean ranks	Sum of ranks	Mann-Whitney U test	p
Chronic periodontitis	30	41.4	1242	123	<0.001
Healthy people	30	19.6	588	-	

## Discussion

The objective of this study was to explore the occurrence of gram-negative oral bacteria, particularly *P. gingivalis*, in the subgingival plaque of individuals diagnosed with CP in comparison to those without any periodontal issues. Periodontal disease includes various conditions affecting the gums, periodontal ligament, cementum, alveolar bone, and other supportive tissues surrounding the teeth [12].

The emergence and progression of active periodontal disease hinge on three primary components: a susceptible host, the existence of pathogenic species, and the absence of beneficial bacteria [15]. The initiation and development of periodontal disease are shaped by the interplay among different genetic, environmental, host, and microbial factors [16,17].

Research has revealed a remarkably wide range of microorganisms associated with periodontal disease. A significant microbial component influencing this ailment is a transition in the composition of oral microflora. Our study's results indicated that the prevalence of *P. gingivalis* in periodontal disease was 23 (78%). This increased occurrence of *P. gingivalis* aligns with observations from other research investigating periodontal disease. These studies consistently report a high incidence of *P. gingivalis* among periodontal patients, ranging from 50.3% to 89.4% across various cases [18-25]. Our research outcomes, in conjunction with these findings, strongly suggest a notable correlation between *P. gingivalis* and the initiation of periodontal disease. This bacterium significantly enhances the likelihood of developing periodontal disease and could be deemed a primary plausible risk factor. The pathogenic characteristics of *P. gingivalis* allow it to invade soft tissues via virulence factors like lipopolysaccharide, fimbriae, and several proteases. Additionally, it can persist even following surgical debridement of periodontal lesions [26,27]. Our investigation also uncovered that the occurrence of *P. gingivalis* was 10 (34%) among individuals without periodontal disease. This bacterium is not limited to individuals with periodontal issues but can also be present in those without periodontal disease, albeit at a lower frequency.

Comparing the findings of our study with existing literature reveals consistent patterns regarding the prevalence of *P. gingivalis* in individuals with periodontal disease. Our study identified *P. gingivalis* in 78% of individuals with CP, aligning with previous research that reported incidence rates ranging from 50.3% to 89.4% in similar populations. This consistency underscores the significant role of *P. gingivalis* in the pathogenesis of periodontal disease. Studies by Slots et al. [28] and Darveau et al. [29] also emphasized that *P. gingivalis* is a key pathogenic species due to its virulence factors like lipopolysaccharides, fimbriae, and proteases, which facilitate tissue invasion and persistence even after periodontal treatment. This reinforces our findings that the presence of *P. gingivalis* is a strong indicator of periodontal disease and supports its classification as a primary risk factor.

Our study also revealed the presence of *P. gingivalis* in 34% of individuals without periodontal disease, which is in concordance with other studies that have documented its presence in healthy individuals, albeit at lower frequencies. This observation highlights the ubiquitous nature of *P. gingivalis* in the oral microbiome, suggesting that its mere presence is not sufficient to cause disease; rather, its pathogenic potential is likely realized in the context of a susceptible host and other environmental and microbial factors. Similar observations have been made by Socransky et al. [30] and Hajishengallis et al. [31], who noted that while *P. gingivalis* is a common inhabitant of the oral cavity; its pathogenicity is significantly influenced by the host's immune response and the overall microbial community composition. Therefore, our findings contribute to the growing body of evidence suggesting that periodontal disease is a multifactorial condition, where the interplay between host susceptibility, microbial virulence, and environmental factors determine disease outcome.

Our study has limitations in both sample size and methodology. Generally, the culture method is considered the gold standard for isolating infectious microorganisms. However, a significant drawback of using culture methods to assess the frequency of periodontal pathogens in subgingival plaque is that only cultivable species and cells are quantified. In other words, species that cannot be cultivated on routine media are not accounted for. Additionally, culture results can be influenced by various factors, including atmosphere, temperature, and cultural media, leading to potential biases. Therefore, alternative methods for detecting bacterial species in subgingival plaque, such as immunofluorescent assays and polymerase chain reaction (PCR), have been found to be more reliable. Further studies with larger sample sizes and improved methodologies are recommended.

## Conclusions

This research suggests that while *P. gingivalis* is commonly found in individuals with periodontal diseases, it is also present in those without such conditions, though in smaller amounts. Therefore, the presence of *P. gingivalis* increases the likelihood of developing periodontal disease and may be considered a significant potential contributor to its onset.
